# Organizational resilience in Puerto Rico's primary care clinics: absorbing, adapting, and transforming under stress

**DOI:** 10.3389/frhs.2026.1886937

**Published:** 2026-08-03

**Authors:** Gabriela Alvarado, Samantha Pérez-Dávila, Alexandra Mendoza-Graf, Marielena Lara, Gabriela Castro, Ilka Ríos, Mariela Torres-Cintrón, Justin Timbie

**Affiliations:** 1 RAND Corporation, Arlington, VA, United States; 2 RAND Corporation, Santa Monica, CA, United States; 3 University of Puerto Rico, Medical Sciences Campus, San Juan, Puerto Rico

**Keywords:** clinic operations, health system resilience, health technologies, health workforce, primary care, Puerto Rico

## Abstract

**Introduction:**

Primary care clinics in Puerto Rico operate under compounding financial, workforce, infrastructural, and environmental constraints that threaten continuity and quality of care. Understanding how these clinics sustain operations under chronic systemic stress and recurrent disasters can yield resilience strategies applicable to safety-net and rural primary care settings more broadly.

**Methods:**

We conducted a cross-sectional qualitative study between May and October 2025 with physicians and clinic leaders at primary care clinics across Puerto Rico, including Federally Qualified Health Centers (FQHCs) and non-FQHC practices. Using purposive sampling, we conducted 25 semi-structured virtual interviews with physicians and executive/medical directors representing all major health regions. Interviews were conducted in Spanish and analyzed using an AI-assisted qualitative coding platform (Muse; inter-rater reliability *κ* = 0.85), followed by matrix framework thematic analysis. We applied a multilevel health system framework alongside organizational and health systems resilience theory to interpret findings.

**Results:**

We identified five themes organized across three resilience levels. At the absorptive level, clinicians expanded their clinical roles, personal accessibility, and informal scope of practice to compensate for specialist shortages, while pursuing high Star Ratings to offset chronically low Medicare Advantage reimbursement. Adaptive resilience included diversifying communication channels, conducting home visits, leveraging municipal resources for transportation and in-home care, and developing multi-modal record systems to maintain care continuity during infrastructure disruptions. Transformative resilience was concentrated in FQHCs, which institutionalized telemedicine, multidisciplinary care teams, and formalized community partnerships as standing responses to recurrent disasters.

**Discussion:**

Puerto Rico's primary care clinics have developed a layered suite of resilience strategies forged under chronic systemic stress and repeated disasters. Federal payment inequities and workforce migration constrain all three resilience levels, with individual clinician burden serving as the primary buffer against system failure. Policy reforms addressing reimbursement parity, workforce retention, and coverage for non-visit-based services are essential to transform individual coping into sustained organizational capacity.

## 1 Introduction

Primary care is a cornerstone of health systems, delivering accessible, continuous, and comprehensive care that supports population wellbeing and addresses both acute and chronic health conditions across the life course (1). In Puerto Rico, primary care operates within a structural and policy environment characterized by chronic underpayment, widespread workforce migration, and fragile clinic operations that long predate recent disasters and public health emergencies (2–4). Reimbursement formulas for federal health care programs have historically paid far below mainland rates, limiting clinics' ability to hire and retain health workers. As a result, many small practices function with thin financial margins, aging infrastructure, and limited administrative capacity.

Hurricane Maria and Irma of 2017 and the COVID-19 pandemic exposed and compounded these vulnerabilities. Infrastructure failures, supply disruptions, displacement of patients and clinicians, and interrupted access to medical records and communications produced abrupt operational shocks for primary care practices across the island (5–7). These were magnified for populations requiring ongoing management of complex chronic conditions, access to specific medications, and caregiver support for daily living (8, 9). While descriptive and policy reports document the macro-determinants of Puerto Rico's health care system constraints and their historical roots (e.g., federal reimbursement inequities, Medicaid underfunding, workforce migration, and Puerto Rico's territorial political status) (2–4, 10, 11), less is known about how primary care clinicians and clinic leaders have developed operational adaptations to sustain patient care amid these compounding challenges, and sustainability beyond acute response phases.

This analysis centers the primary care clinic as the operative unit through which systemic pressures are experienced and managed, providing perspectives often missing from analyses that rely primarily on administrative data or provider exit counts. Drawing on a qualitative approach informed by a multi-level health systems framework and organizational resilience theory (12, 13), our objective was to document how clinics maintain continuity and quality of care under overlapping financial, workforce, infrastructural, and environmental constraints, and to distill lessons applicable to safety-net and rural primary care settings facing analogous systemic stresses. These frameworks were selected because they capture the layered pressures Puerto Rico's primary care clinics face and provide a lens for understanding how clinics respond to and recover from stress. Resilience theory, in particular, allowed us to move beyond documenting system failures toward examining the active strategies clinicians and clinic leaders deploy to sustain care.

## 2 Methods

### 2.1 Design

This qualitative study forms part of a larger mixed-methods study focusing on care for older adults in Puerto Rico living with Alzheimer's disease and related dementias (AD/ADRD), with a focus on the operational experience of primary care clinics. We adopted a multilevel model of health system change as our conceptual anchor (13, 14), tracing how pressures and adaptive responses emerge across patient, team, organizational, and policy levels. To interpret clinic responses, we drew on resilience theory, specifically Turenne et al.'s absorptive, adaptive, and transformative typology and Lengnick-Hall and Beck's model of adaptive fit and robust transformation (12, 14, 15).

Absorptive resilience refers to the capacity of clinics to maintain essential functions while absorbing shocks using existing resources. Adaptive resilience reflects the ability of clinics to modify routines, workflows, and relationships to maintain continuity when resource constraints exceed individual capacity. Transformative resilience represents the most advanced level of response, where repeated crises lead clinics to reconfigure systems for long-term sustainability.

The study was approved by the Institutional Review Board of each author's institution, and all methods and results are reported here according to the Standards for Reporting Qualitative Research (SRQR) Guidelines (16).

### 2.2 Study setting and recruitment

Between May 2025 and October 2025, we conducted 25 semi-structured interviews with physicians at Federally Qualified Health Centers (FQHCs) (*n* = 6) and physicians in non-FQHC primary care clinics (*n* = 19), purposively sampled to represent diverse perspectives on providing care to individuals living with Alzheimer's disease and related dementias (AD/ADRD). FQHCs are community-based primary care clinics that receive federal grant funding to deliver comprehensive care to medically underserved populations regardless of ability to pay (17). This designation confers benefits such as enhanced Medicare/Medicaid reimbursement, eligibility for federal capital and operational grants, participation in drug pricing programs, and formal affiliation with primary care associations that provide technical assistance and emergency-preparedness support.

We contracted recruitment services from a private firm specializing in research involving clinicians that conducted outreach to clinics in Puerto Rico via email, text, and phone to identify and screen potential participants. Eligibility criteria included being an executive director, medical director, or practicing physician at a primary care clinic and having been at their current organization for at least 3 years.

### 2.3 Sample and data collection

Researcher triads conducted 25 one-hour virtual interviews via ZoomGov in Spanish; one team member led the interview and the second served as question prober to support follow up questions, while the third took notes. Participants provided verbal consent and agreed to audio recording prior to starting the interview, which followed a semi-structured guide (Table 1). Audio recordings were used for later transcription. Participants received compensation for their time and will receive copies of all reports and publications.

**Table 1:** Interview domains of inquiry and sample questions.

Domain of inquiry	Sample question(s)
Current clinic operations	- What are some of the operational strengths of your clinic? - What do physicians say is challenging or could be better about practicing at your clinic?
Strategies, successes, and challenges in providing care to patients	- Overall, how easy or difficult is it for your Alzheimer's patients to obtain care from your clinic when they need it?^a^
Care coordination processes	- How do physicians learn about the care patients receive from other specialists?
Supports available for patients and caregivers	- What kind of support is your clinic able to offer patients? - What are the most common caregiver needs?
Effects of natural disasters on healthcare access and delivery	- How did Hurricane Maria affect your clinic? - How have Puerto Rico's more recent disasters impacted care for your patients?

a Given the study’s focus on care for older adults with Alzheimer’s disease and related dementias (AD/ADRD), select questions probed AD/ADRD-specific care experiences; other questions addressed clinic operations and patient care more broadly.

### 2.4 Analysis

Audio recordings were transcribed in their original language (Spanish) for analysis. A random sample of transcripts (*n* = 6) were used to develop a codebook. Each sample transcript was coded sequentially by team members through open and *in vivo* coding to assess for code saturation and by the 4th interview code saturation was achieved, which we defined as an increase in the number of new codes of less than 5% (18). An additional transcript was selected to verify saturation. We then proceeded to collaboratively and iteratively refine the list of codes into a codebook, informed by the health system multi-level framework and concepts of organizational resilience (13, 14).

We collaboratively coded a purposively selected transcript—chosen based on its richness and detailed descriptions of operational issues in primary care clinics in Puerto Rico. We then used this coded transcript to set up an inter-rater reliability test to leverage an AI-coding tool called Muse (19, 20). Following an approach similar to human-coder trainings conducted in Dedoose, we used a single, content-rich transcript that exemplified common and complex coding contexts. This allowed multiple rounds of codebook refinement and model retraining without introducing potential memory bias or inconsistency. Although inter-rater reliability was based on a single transcript, it captured all but one code from the full codebook (the recommendations code), offering an efficient calibration dataset before applying the final model to the complete corpus. After three rounds of codebook refinement and model training, we achieved a Kappa score of 0.85, which represents a strong level of agreement between human coding and the AI-coding (21), after which we felt confident using Muse for independent coding.

Coded data from Muse were exported and analyzed for themes using a matrix framework (22). We organized these themes along the theories that guided our analytic strategy (15). No generative AI was used to construct themes, however we did leverage the Muse platform to help identify quotes to support the themes. Quotes included in this paper were translated by one team member (GA), a native Spanish speaker, and reviewed for accuracy by other Spanish speakers on the team.

Our predominantly Latin American research team brought shared linguistic and cultural perspectives that enhanced sensitivity to participants' accounts while prompting reflection on our tendency to emphasize strengths over structural deficits; we addressed this tension through regular team discussions.

## 3 Results

Our analytic sample included 25 clinicians and clinic leaders representing a range of primary care organizations across Puerto Rico (Table 2). Respondents were nearly evenly split by gender and included both physicians and executive or medical directors with varied tenure, from early-career to more than 20 years at their current clinic. Participating clinics were distributed across all seven of Puerto Rico's Department of Health health regions, with roughly 70% located outside the San Juan metropolitan area. Given that population and health infrastructure in Puerto Rico are heavily concentrated in the San Juan metropolitan area, this distribution reflects a deliberate effort to capture perspectives from smaller and more rural clinics that are otherwise underrepresented in health services research on the island.

**Table 2:** Characteristics of clinic representatives participating in interviews.

Respondent characteristics	Primary care clinic (non-FQHC) respondents (*n* = 19)	FQHC respondents (*n* = 5)^a^	All respondents (*n* = 24)
Gender, *n* (%)			
Female	9 (47%)	2 (40%)	11 (46%)
Male	10 (53%)	3 (60%)	13 (54%)
Role at clinic, *n* (%)			
Executive director or medical director (including practicing physicians)	5 (26%)	5 (100%)	10 (42%)
Physician	14 (74%)	0 (0%)	14 (58%)
Years at clinic, *n* (%)			
≤5 years	3 (16%)	1 (20%)	4 (17%)
6–10 years	5 (26%)	2 (40%)	7 (29%)
11–20 years	6 (32%)	0 (0%)	6 (25%)
≥21 years	5 (26%)	2 (40%)	7 (29%)
Clinic location, *n* (%)			
San Juan metropolitan area	6 (32%)	1 (20%)	7 (29%)
Outside San Juan metropolitan area	13 (68%)	4 (80%)	17 (71%)
Clinic affiliation, *n* (%)			
Independent physicians association (IPA)	16 (84%)	0 (0%)	16 (67%)
Medical group	2 (11%)	0 (0%)	2 (8%)
Private practice	0 (0%)	0 (0%)	0 (0%)
Federally qualified health center (Centros 330)	0 (0%)	5 (100%)	5 (21%)
Other	1 (5%)	0 (0%)	1 (4%)
Number of physician FTEs at clinic, *n* (%)			
1	5 (26%)	0 (0%)	5 (21%)
2–4	7 (37%)	0 (0%)	7 (29%)
5–9	5 (26%)	0 (0%)	5 (21%)
≥10	2 (11%)	5 (100%)	7 (29%)

a Only 5 of the 6 FQHC participants represented in this table.

From our analysis we constructed five themes which we organize along three levels reflecting a progression of resilience capacities: from absorptive practices that buffer stress within existing resources, to adaptive process modifications that extend clinic reach, and finally to transformative changes that institutionalize new care models (Table 3).

**Table 3:** Resilience strategies and themes resulting from operational challenges at the primary care level in Puerto Rico.

Theme	Operational challenge	Operational impact	Resilience strategies
Clinicians expanded their training, clinical roles, and personal accessibility to address systemic workforce shortages	Workforce shortages: clinician out-migration to the mainland and aging of the current PR workforce	Long wait times for appointments and increased physician workload	Absorptive resilience: - PCPs act as specialist as a stopgap measure - PCPs increase their level of accessibility by sharing email and phone contact information with patients - Warm hand-offs to specialists
Clinics pursue performance-based incentives, such as high Star Ratings, to offset chronically low reimbursement and sustain financial viability	Reimbursement rates: Inadequate reimbursement (40% below U.S. mainland for Medicaid Advantage) and high claims denials, coupled with high inflation	Undermines ability to provide comprehensive, continuous, and patient-centered care	Absorptive resilience:
- Prioritizing clinical workflows that drive high Star Ratings - Staff take on additional documentation and paperwork duties
Care teams adjust outreach and workflows to offset coverage gaps in transportation and home-based support	Coverage limitations: Lack of coverage for transportation, in-home personal care, or adult day centers	Caregiver exhaustion and lack of transportation can lead to missed appointments and lapses in care	Adaptive resilience:
- Care teams make home visits to patients without transportation - Leveraging municipality-based resources such as “ama de llaves” and transportation services
Clinics developed multi-modal strategies and infrastructure redundancies to preserve access to information and improve care coordination	Infrastructure and technology: prevalence of paper-based systems and EHRs with limited EHR interoperability which are further compromised by frequent power outages	Fragmented records and reliance on caregivers and patients to transfer information across providers	Adaptive resilience:
- Care coordination workflows include faxing patient information - Patients and caregivers act as liaisons in their own care, bringing documents back and forth between providers - Redundancy protocols for outages
Clinics institutionalized telemedicine, multidisciplinary coordination, and community partnerships as responses to recurrent disasters and environmental stressors	External factors: contracting economy, high social need, and demographic transition	Increased patient need and isolation impacts ability to maintain continuity of care	Transformative resilience:
- Permanent telemedicine integration - Established disaster protocols and back-up generators - Case managers and social workers connect patients with services

### 3.1 Absorptive resilience: withstanding systemic stress

#### 3.1.1 Theme 1: clinicians expanded their training, clinical roles, and personal accessibility to address systemic workforce shortages

Clinicians described a severe provider shortage across all sectors of Puerto Rico's health system, explaining that many colleagues migrated to the U.S. mainland in search of better pay, working conditions, and stability, leaving remaining clinicians to contend with shrinking clinical and referral capacity. These provider shortages were particularly acute for specialists and were exacerbated by natural disasters.

“I understand that after Maria and the earthquakes there was a brain drain of providers, which resulted in the current lack of services we have now. I have lots of colleagues who said I’m going to the U.S. and would say they would only go two or three years while things get back to normal, but never came back”—Medical Director

Clinicians reported that these losses continue to translate into long waits for appointments, overbooked schedules, and reduced time with each patient. This rising workload also negatively impacts the quality of care provided to patients.

“Many times it's overload and I see that it is affecting things at that level too – sometimes doctors see patients fast and miss things, they write wrong prescriptions, they are tired …”—Medical Director

Despite mounting strain, physicians across clinics absorbed these shocks by expanding their scope of practice and availability to patients. Without specialists, primary care doctors took on complex cases and sought informal training to fill gaps. They also mitigated specialist shortages by personally coordinating warm handoffs to trusted colleagues.

“Most times I have to step in, I have to call. I have a neurologist friend who I call and provide them the information and patient's phone number and they usually try to fit the patient in.”—Medical Director

Physicians often described instances of *doing more with less* to keep patients from falling through the cracks; compensating by expanding their scope of work beyond medical visits to include community outreach, laboratory coordination, and direct communication with caregivers to troubleshoot daily challenges. This often meant sharing personal phone numbers or creating direct messaging channels so patients/caregivers could reach their physician for quick guidance.

“They all have my cell number – for better or worse. It's like we say, once I make the diagnosis, I create sort of a love triangle between the patient, the caregiver, and myself, and they all have my number. That way I can maybe help a little more quickly.”—Medical Director

These coping practices, though effective, depend on individual vocation rather than structural support. One clinician reflected on their personal commitment to their profession and emphasized that “I love what I do, I was born for this, and I consider my patients my family”. Yet as responsibilities accumulated, many described exhaustion and emotional depletion, creating a reinforcing cycle where each departure increases the burden on those who remain.

“We doctors are burned out, truly. Extreme burnout I imagine. There are no doctors. We are working with a number of patients that goes beyond what a doctor can handle.”—Primary Care Physician

#### 3.1.2 Theme 2: clinics pursue performance-based incentives, such as high star ratings, to offset chronically low reimbursement and sustain financial viability

Clinics made adjustments aimed at keeping their practices solvent under chronically low reimbursement. A central strategy was prioritizing quality metrics, especially achieving high Star Ratings under Medicare Advantage (MA), to secure bonus payments that could help buffer operating costs and partially compensate for low base rates.

“You know that the medical fees in Puerto Rico are very low, right? The lowest paying ones are in Mississippi and Louisiana, and we are [paid] 60 percent below that.”—Medical Director

Clinicians portrayed reimbursement as insufficient and unstable. Particularly frustrating were health plans’ profiling of physician-level spending that could trigger cuts to capitation payments for “high-cost” providers—who may simply be caring for high-need patients. Independent Physician Associations (IPAs) function as financial intermediaries that determine final payments, further distancing clinical work from predictable revenue streams.

“When you’re part of an IPA, you sign a contract and you’re bound by what they call the rules of the game. And the downside of that is that one of the clauses says you should be a doctor who doesn't spend too much, because if you start ordering test or certain medications that are more expensive, then the IPA will say, ‘Look doctor, that patient is spending too much and you should make an adjustment in how you manage them.”—Primary Care Physician

Clinics must also absorb administrative labor such as prior authorizations, denials, and compliance reporting, all of which are essential to keep payments flowing but are rarely reimbursed. Physicians noted that the burden is heightened by Puerto Rico's concentrated MA market and low payment rates, which give health plans outsized control and limit providers' ability to contest denials or negotiate terms.

“If they don't like that [diagnosis] code, then they deny the bill and don't pay me. Or they tell me I have to review the code. In my case, I do the math and realize, honestly, it's more trouble than it's worth, because in Puerto Rico those plans pay virtually nothing, and if I’m sending a bill for only ten dollars and on top of that you don't even pay me.”—Medical Director

Over time, these documentation requirements demand redirecting attention from patient care to reimbursement-driven metrics, especially meeting follow-up care deadlines imposed by Star Ratings measures. Several participants described at length the intense pressure providers feel to meet certain measures such as follow-up visits 7 days after an emergency room visit, which can be logistically challenging, especially when there are no integrated record systems.

“I think the health plans demand a lot in regards to meeting the Star Ratings; they also demand things that are beyond our control as doctors [such as patients’ access to reliable transportation to attend visits].”—Primary Care Physician

The cumulative effect is operational strain and relational tension with clinicians expressing frustration with low pay and intensive oversight. To persist, clinics rely on practical workarounds. Staff with capacity absorb billing and authorization tasks to secure timely payment, which as one provider noted, “consumes time that no one pays for”.

### 3.2 Adaptive resilience: adjusting workflows and processes

#### 3.2.1 Theme 3: care teams adjust outreach and workflows to offset coverage gaps in transportation and home-based support

Participants highlighted major coverage gaps in transportation, explaining that insurance plans limit trips, do not have enough available vehicles, or require advance scheduling. One physician explained that, “[rides] cannot be secured from one day to the next”, which means that if a patient is given a last-minute specialist appointment, they cannot attend. Likewise, few health plans cover in-home personal-care services or adult day centers—services that participants viewed as critical for Puerto Rico's rapidly aging population. When family members cannot reliably bring patients to appointments, or when home assistance is not covered, primary care teams described expanding their reach beyond the clinic walls to ensure continuity. Electronic prescribing, phone calls, and telemedicine visits were common strategies.

“There are patients who live in the countryside and have trouble getting here […], but sometimes they prefer to do a phone or video consultation, which I can offer.”—Primary Care Physician

Clinics also set up multiple, redundant communication lines so that patients and caregivers could reach the care team quickly through whichever channel worked, including telephone, WhatsApp, or a designated referral line.

“We have two ways to communicate because our mission is just that – we’re here for them. We have about six phone lines: there is the general line, a WhatsApp line they know very well, we also have Facebook pages that sometimes work for communication […] and we have a special line just for referrals.”—Medical Director

When virtual contact was insufficient, some physicians conducted home visits, or sent laboratory technicians for in-home laboratory draws and testing. Unlike in many mainland U.S. practices, these home visits do not carry additional reimbursement, making the effort financially and logistically burdensome. Still, physicians described them as a moral and professional obligation to patients who would otherwise go without care.

“We are always there to help everyone, I have no problem with that. Even when they can't come to the office, I visit bedridden patients in their home.”—Medical Director

One specific clinic went even further, informally turning staff into *ad-hoc* drivers when plan-sponsored transportation failed.

“If we see that [a patient] has missed multiple appointments, sometimes we can arrange to have lab tests drawn at home. Sometimes, in addition to coordinating with their health plan, if too much time passes, we try to arrange for a staff member from the office to go pick them up, but that's something more limited we’ve been doing.”—Primary Care Physician

Beyond transportation, clinics regularly negotiated with municipal governments to secure scarce *amas de llave* (publicly funded in-home aides) for cognitively impaired or functionally limited patients. Some formalized these linkages through community outreach programs that identify social needs and broker ties with external agencies to provide services.

“Sometimes we get in touch with the municipalities. Municipalities sometimes have amas de llaves available for residents, but there aren't many spots for that. And usually we have to be in constant contact with the municipalities to be able to have our patients access those services.”—Medical Director

Overall, clinics compensated for coverage and infrastructure gaps by diversifying communication channels, repurposing staff roles, and integrating local resources into patient care.

#### 3.2.2 Theme 4: clinics developed multi-modal strategies and infrastructure redundancies to preserve access to information and improve care coordination

Primary care clinics described a form of operational resilience built around hybridizing electronic and paper-based information systems to ensure information access and continuity when connectivity or interoperability failures occur. Limited electronic health record (EHR) interoperability meant that digital records often did not travel smoothly across settings, impacting how services are delivered.

Further, clinics operate in an environment where infrastructure disruptions are routine: power outages, intermittent internet connectivity, server and equipment damage from humidity or voltage fluctuations, and the high costs of rebuilding after natural disasters make fully digital workflows difficult to sustain consistently. This compelled some clinics to combine electronic tools with paper-based/manual records to keep information and basic coordination.

“And from then on [after the hurricane], even though I had electronic records, for everyone I now make both an electronic and a paper record. I mean, I still keep paper folders, in addition to the electronic records.”—Primary Care Physician

Clinics described that sharing patient information often requires faxing documents between providers and facilities, and that administrative tasks such as faxing, scanning, and manual data entry, expand workload while limiting the time available for direct patient care. To maintain coordination when electronic systems failed, clinicians intentionally enlisted caregivers and patients as part of the information-transfer process. Physicians described asking family members to carry documents, test results, and referral paperwork between providers. Primary care physicians also described personally coaching patients and caregivers through referral processes, contacting specialists directly, and guiding families on how to share visit information.

“We always ask the family member: give this document [to the doctor] and please ask them to prepare a note from the visit so that I can know what happened and we can stay in communication.”—Medical Director

While these hybrid workflows allowed for some continuity of care, clinicians noted persistent obstacles linked to the administrative realities of reimbursement and compliance. Limited resources constrain clinics' ability to purchase and maintain sophisticated cloud-based EHRs, while insurers' documentation and reporting requirements demand repeated manual data entry. Compliance gaps also occur when specialists do not return visit reports or when patients forget to deliver paper forms back to the clinic, leaving records incomplete and hampering coordination.

### 3.3 Transformative resilience: re-configuration for sustainability

#### 3.3.1 Theme 5: clinics institutionalized telemedicine, multidisciplinary coordination, and community partnerships as responses to recurrent disasters and environmental stressors

Repeated exposure to hurricanes, earthquakes, and public health emergencies resulted in a shift from temporary workarounds to sustainable approaches. Clinics across Puerto Rico described how strategies first improvised after Hurricane María, such as phone-based consultations and other low-tech communication methods used when infrastructure collapsed, have gradually evolved into more formal mechanisms for remote care. These initial measures laid the groundwork for rapid adoption of video- and platform-based telehealth during the COVID-19 pandemic, which many participants identified as the moment when remote care became normalized. Telehealth visits are perhaps the most visible example of this transformation.

“There are many clinics that started doing telemedicine after the disasters. During the pandemic, since there was the challenge that no one could go into clinics because everything was locked down, everything turned into telemedicine. And then it became hybrid, like a mix, with in-person for those who wanted it, and we also continued with telemedicine.”—Medical Director

However, telemedicine represents only one dimension of this broader institutionalization of resilience. FQHCs were the primary sites of transformative change, with long-standing multidisciplinary care models expanding in scope to address social and logistical barriers. Providers explained that psychologists, case managers, and social workers take on broader roles in home-based visits, functional assessments, and caregiver coordination—reflecting how clinics receiving federal grant funding institutionalize outward-facing, community-embedded care.

External partnerships have also been formalized and codified into emergency plans. Some FQHCs drew on resources available through their primary-care association to secure satellite phones and sustain communication with peer clinics during the island-wide outages that followed Hurricane María. Other FQHCs forged standing agreements with municipal governments to coordinate emergency transport, deploy mobile units to reach isolated communities, and access publicly funded caregivers when insurer benefits fell short. These collaborations illustrate how community partnerships have moved from crisis improvisation to standard operating procedure, though primarily within FQHC settings.

Federal grants and philanthropic initiatives have helped fund some of these upgrades, yet ongoing inflation and workforce shortages continue to threaten long-term sustainability. One physician underscored the importance of preparing for future disasters.

“The experience of having lived through these disasters taught us that we need to prepare our practice for future disasters. If we’re not prepared, we’ll end up falling into the same situation again.”—Primary Care Physician

## 4 Discussion

In this qualitative analysis of 25 interviews of physicians and physician executives in Puerto Rico we found that primary care clinics sustained care delivery amid numerous operational challenges by leveraging a continuum of resilience capacities: absorptive, adaptive, and transformative. This typology provides a useful framework for interpreting how organizations learn and evolve under chronic stress. Organizational resilience in resource-constrained settings is not a single event, but an ongoing process of learning and recalibration. Although prior studies have documented long-standing payment inequities and workforce migration in Puerto Rico and their impacts on patient care (2, 4, 5, 7), these challenges occur within a broader socioecological context of poverty, deficient infrastructure, and limited political autonomy (23)—all of which shape the resilience patterns we found.

At the absorptive level, clinics stabilized operations primarily through individual commitment and vocational effort, with physicians expanding their roles and accessibility to compensate for specialist or staff shortages. These behaviors exemplify what Lengnick-Hall and Beck describe as adaptive fit, which are responses that buffer performance without altering underlying structures (14). While vital for short-term continuity, this dependence on personal labor rather than institutional support underscores the fragility of Puerto Rico's health system. As the Puerto Rico Healthcare Workforce Study reports, 47 percent of physicians in active practice are 60 years or older, the highest share in any U.S. jurisdiction, and nearly half plan to retire within 5 years (24). Ethnographic accounts also demonstrate how provider migration, precarious insurance structures, and political uncertainty intersect to produce widespread distress among both patients and clinicians (25). Without systematic retention and replacement strategies, the absorptive layer of resilience is at risk, and because absorptive capacity is the foundation on which adaptive and transformative capacities are built, its erosion constrains the entire resilience continuum. As long as individual clinician burden remains the primary buffer against system failure, clinics are structurally prevented from advancing to the higher resilience levels where durable, policy-relevant change is possible. These findings echo previous analyses noting the difficulty of institutionalizing change without reimbursement structures that enable caregiver education, care planning, and the coordination time required for complex patients, including those with Alzheimer's disease and related dementias (4, 26).

At the adaptive level, clinics moved from individual coping to redesigning organizational workflows—an expression of what Blanchet et al. describe as system governance and adaptive capacity: the ability to modify processes to sustain functionality under stress (15). Comparable patterns have been documented in rural U.S. clinics and systems responding to Hurricane Katrina (27). Yet, unlike many mainland settings, Puerto Rican clinics face federally imposed funding constraints that shape what feasible adaptation means in practice. Territorial policy design continues to limit parity in Medicaid and Medicare reimbursement rates (10, 11), directly influencing clinic viability, hiring, and the ability to invest in non-visit-based services essential to coordination and dementia care.

Lastly, transformative resilience captures the institutionalization of previously improvised strategies. Repeated exposure to hurricanes, earthquakes, and public health emergencies catalyzed permanent integration of telemedicine, multidisciplinary teams, and community partnerships into everyday operations. Organizations formalized disaster response protocols, introduced infrastructure redundancies such as solar power and backup communications, and embedded case managers and social workers into clinical teams. In alignment with organizational theory, these actions embody organizational learning by shifting from reactive adaptation to proactive transformation. These findings emphasize that capacity for transformation is uneven across organizations; changes of this magnitude were concentrated primarily within Federally Qualified Health Centers, which had pre-existing multidisciplinary infrastructure and institutional resources that positioned them to evolve more rapidly than smaller private practices. This greater capacity for transformative resilience reflects structural access to resources not available to independent, non-FQHC practices. Broadening this capacity could involve extending federal safety-net funding streams and technical assistance to non-FQHC clinics serving comparably vulnerable populations or supporting regional consortia that allow smaller practices to pool resources for emergency preparedness and disaster response.

Transitions and movement between the stages of resilience appeared most clearly where repeated exposure to disruption prompted retrospective, intentional reflection rather than mere repetition of coping behavior: participants described how improvised, absorptive-level workarounds developed during Hurricane María were revisited and consciously formalized into standing protocols after subsequent disasters, suggesting a cumulative, experience-based learning process rather than a single linear progression. This pattern echoes organizational learning frameworks in which reflection on past crises can evolve into changes to underlying structures and assumptions (28).

Prior analyses outside of the U.S. have similarly noted that most health system resilience research emphasizes absorptive and adaptive capacities, with transformative resilience such as changes in structure, governance, or legitimacy, seldom examined (29). By demonstrating how recurring stressors prompted enduring organizational change, our findings extend this literature and mirror global recommendations emphasizing infrastructure strengthening, continuity of care, and proactive rather than reactive planning (30). As a U.S. territory, structural dependency and limited autonomy (2–4) shape the boundaries and the possibilities of resilience in the island's health system. For example, Medicare Advantage enrollees in Puerto Rico receive substantially lower quality of chronic-disease management and preventive care than comparable Hispanic beneficiaries in the mainland U.S (31). These findings resonate with a recent regional Commission on primary care resilience across Latin America and the Caribbean, which modeled the substantial health and economic costs of underinvesting in primary care resilience and concluded that strengthening primary care and building health system resilience are not competing priorities but mutually reinforcing goals (32).

Translating these findings into policy requires action at each resilience level: stabilizing absorptive capacity through workforce retention incentives, and burnout mitigation; strengthening adaptive capacity by expanding coverage for transportation and home-based services, simplifying prior authorization, and investing in interoperable disaster-ready data systems; and anchoring transformative resilience by embedding crisis lessons into everyday governance and achieving reimbursement parity (2, 10). Taken together, these directions treat resilience as a continuous process of system maintenance rather than an emergency response.

While this study offers insight into operational resilience in Puerto Rico's primary care clinics, several limitations warrant consideration. First, our findings derive from a purposive sample of physicians and clinic leaders and may not capture experiences across all regions or clinic types. Because of our sample size, and small number of FQHC respondents, we did not conduct formal subgroup analyses by different clinic or respondent characteristics. Additional limitations include the cross-sectional design, and the strengths-based orientation that may understate persistent structural deficits experienced by the most vulnerable populations.

Despite these constraints, the Puerto Rican experience offers valuable transferable lessons. Rather than an exceptional case of vulnerability, Puerto Rico represents a compelling case study in how clinics adapt to chronic adversity in ways that resonate with safety-net and rural primary-care practices across the mainland U.S. By shifting the narrative from fragility to continuous adaptation and learning, Puerto Rico's primary care sector demonstrates pathways through which under-resourced systems can transform under pressure, a message equally relevant for other regions confronting the dual realities of constrained resources and increasing environmental stressors.

## Data Availability

The datasets presented in this article are not readily available because small sample of qualitative data not available for sharing per IRB. Requests to access the datasets should be directed to jtimbie@rand.org.
